# Clinical Characteristics of Anti-Synthetase Syndrome and Variables Associated with Interstitial Lung Disease and Mortality: A Retrospective Cohort Study

**DOI:** 10.3390/jcm12216849

**Published:** 2023-10-30

**Authors:** Tulaton Sodsri, Tananchai Petnak, Pintip Ngamjanyaporn

**Affiliations:** 1 Chakri Naruebodindra Medical Institute, Faculty of Medicine Ramathibodi Hospital, Mahidol University, Samut Prakan 10540, Thailand; tulatui21@gmail.com; 2 Division of Pulmonary and Pulmonary Critical Care Medicine, Department of Medicine, Faculty of Medicine Ramathibodi Hospital, Mahidol University, Bangkok 10400, Thailand; 3 Division of Allergy, Immunology and Rheumatology, Department of Medicine, Faculty of Medicine Ramathibodi Hospital, Mahidol University, Bangkok 10400, Thailand; pintip.nga@mahidol.ac.th

**Keywords:** anti-synthetase syndrome, autoantibodies, interstitial lung disease, mortality, idiopathic inflammatory myositis

## Abstract

Anti-synthetase syndrome (ASS) is a rare autoimmune disease. Since the knowledge of ASS remains limited, we conducted the retrospective study aiming to describe clinical characteristics and identify variables associated with interstitial lung disease (ILD) and mortality among patients with ASS. Patients diagnosed with ASS from January 2013 to October 2022 were included. Patient demographics, clinical manifestations, myositis auto-antibody profiles, HRCT findings, and laboratory tests were collected. Variables associated with mortality risk and ILD were evaluated using the Cox proportional hazards model and the logistic regression model, respectively. A total of 82 patients with ASS were included. Clinical manifestations included arthritis (57%), Raynaud’s phenomenon (32%), mechanic’s hands (29%), fever (26%), and myositis (17%). The myositis auto-antibody profiles included anti-PL-7 (29%), anti-Jo-1 (27%), anti-EJ (17%), anti-PL-12 (16%), and anti-OJ (11%). ILD was observed in 64 patients (78%). Among patients with ILD, 21 initially presented with ILD before developing other ASS clinical manifestations, 29 simultaneously presented with ILD and other symptoms, and 14 had isolated ILD throughout follow-up. Overall, 6 patients presented with rapid-progressive ILD. With a median follow-up time of 2.5 years, mortality was observed in 10 patients (12.2%). Factors associated with mortality included increased lymphocyte counts (adjusted HR, 0.74; 95% CI, 0.61–0.91; *p* < 0.01), isolated ILD (adjusted HR, 9.59; 95% CI, 1.52–60.61; *p* = 0.02) and the presence of anti-Ro52 antibodies (adjusted HR, 0.14; 95% CI, 0.02–0.93; *p* = 0.04). Factors associated with ILD included age (adjusted OR, 1.10; 95% CI, 1.03–1.18; *p* = 0.01), presence of anti-Ro52 antibodies (adjusted OR, 17.92; 95% CI, 2.13–138.68; *p* = 0.01), and presence of arthritis (adjusted OR, 0.09; 95% CI, 0.01–0.75; *p* = 0.03). Our study demonstrated a favorable overall mortality rate among ASS patients.

## 1. Introduction

Anti-synthetase syndrome (ASS) represents a distinct subset within the idiopathic inflammatory myopathies (IIMs) spectrum, affecting various organ systems including the lungs, skin, joints, and muscles. Diagnosis of ASS requires the identification of antibodies against aminoacyl-transfer RNA (tRNA) synthetase incorporated with clinical symptoms including fever, arthritis, interstitial lung disease (ILD), Raynaud’s phenomenon, and mechanic’s hands. The establishment of an ASS diagnosis is guided by two diagnostic criteria, Connors’ and Solomon’s criteria [1,2]. According to Connors’ criteria, a diagnosis of ASS requires the presence of an anti-aminoacyl (tRNA) synthetase antibody along with at least one clinical manifestation, whereas Solomon’s criteria require the presence of two or more clinical manifestations.

In the spectrum of antibodies associated with ASS, anti-Jo-1 is not only the most common antibodies but also the first antibody identified in this autoantibody family. The remaining antibodies include anti-PL-7, anti-PL-12, anti-EJ, anti-OJ, anti-KS, anti-Zo, and anti-HA [3,4]. Notably, patients presenting with various anti-synthetase antibodies may manifest a spectrum of distinct clinical manifestations. For instance, patients with anti-Jo-1 antibodies typically exhibit an initial clinical presentation characterized by arthritis and are associated with a more diffuse clinical phenotype. In contrast, patients with anti-PL-7, anti-PL-12, and anti-OJ antibodies often manifest with isolated ILD as a prominent feature [5,6].

ILD is common in ASS and often appears as the initial clinical manifestation [7]. The pathogenesis of ILD in ASS is complex, including a multifaceted interplay of immune-mediated inflammatory processes, pulmonary fibrosis, and remodeling of the lung parenchyma. Furthermore, ILD can result in significant respiratory impairment and eventually respiratory failure. Even though rapid-progressive ILD (RP-ILD) is relatively uncommon, respiratory failure and increased mortality risk were observed.

The prognosis for ASS varies significantly based on various variables, including the severity of the disease and the presence of specific autoantibodies. Some studies indicated that the 5-year survival rate for anti-Jo-1-positive patients is significantly higher when compared with those with negative anti-Jo-1 (75% versus 90%) [8]. The presence of ILD is associated with a worse prognosis in ASS [6]. Other poor prognostic factors include older age, male gender, and the presence of cardiac or renal involvement [9].

Since the knowledge of ASS remains limited and controversial, we conducted a retrospective study to describe clinical characteristics and identify variables associated with the presence of ILD and mortality among patients with ASS.

## 2. Materials and Methods

A single-center retrospective study of ASS patients was conducted at Ramathibodi Hospital, Bangkok, Thailand. The study was approved by the Faculty of Medicine Ramathibodi Hospital Institutional Review Board (COA. MURA2023/221). Patients who underwent a myositis-specific antibody (MSA) panel between January 2013 and October 2022 were screened to identify positive results for ASS-associated autoantibodies, including anti-Jo-1, anti-PL-7, anti-PL-12, anti-OJ, and anti-EJ. The MSA test used in general service at our institute was EUROIMMUN immunoblots^®^. The panel consists of anti-Jo-1, anti-PL-7, anti-PL-12, anti-EJ, anti-OJ, anti-SRP, anti-Mi2, anti-SAE, anti-NXP2, anti-MDA5, anti-TIF1γ, anti-HMGCR, anti-cN-1A, anti-PM-Scl, anti-Ku, and anti-Ro52.

For patients with positive ASS-associated autoantibodies, the electronic medical records, chest X-ray, and HRCT were comprehensively reviewed to ascertain the presence of ASS and its associated manifestations at the time of diagnosis and throughout the follow-up period. These included arthritis, Raynaud’s phenomenon, mechanic’s hands, fever, ILD, ILD pattern, rash, muscle weakness, and myositis. All clinical manifestations were assessed and documented by rheumatologists and/or pulmonologists, whereas the diagnosis of ILD was confirmed through the HRCT. RP-ILD was defined as the deterioration of ILD occurring within three months of the onset of respiratory symptoms. The diagnosis of myositis was based on elevated serum creatinine kinase or aldolase levels, with or without histopathological confirmation.

Patients were included in the study if they met the criteria of being more than 18 years old and having ASS [1]. The patient demographic data, clinical manifestations, laboratory findings, and radiographic findings were recorded. The objective of this study was to identify variables associated with ILD and mortality among ASS patients. The diagnosis of ASS-associated and overlapping connective tissue diseases adhered to the international diagnostic criteria, including the 2017 EULAR/ACR classification criteria for IIM, the 2016 ACR/EULAR classification criteria for primary Sjogren’s syndrome, the 2019 EULAR/ACR classification criteria for systemic lupus erythematosus (SLE), the 2013 ACR/EULAR classification criteria for systemic sclerosis, and the 2010 ACR/EULAR classification criteria for rheumatoid arthritis.

Continuous data were summarized by the mean ± SD or median (interquartile range, IQR) based on data distribution, whereas categorical data were reported as frequency and percentage. Chi-square or Fischer’s exact test were used to compare categorical variables and the independent sample *t*-test or Mann–Whitney U test for comparing continuous variables between two groups, where appropriate. A univariate Cox proportional hazards model was performed to evaluate mortality risk, whereas the logistic regression model was used to assess variables associated with ILD. Variables which had *p*-values of ≤0.1 were included in the multivariate Cox proportional hazards regression or multivariate logistic regression model using the backward method, as appropriated. The cumulative survival rate was demonstrated using the Kaplan–Meier survival curve. A *p*-value of less than 0.05 was considered statistically significant.

## 3. Results

### 3.1. Patient Characteristics and Clinical Manifestations

Between January 2013 and October 2022, a total of 1666 MSA panels were performed. Among these, 123 patients tested positive for ASS autoantibodies. A total of 41 patients were excluded due to the presence of isolated ASS autoantibodies without clinical manifestations that satisfied Connors’ criteria. Consequently, 82 patients were finally diagnosed with ASS based on Connors’ criteria. Of the 82 ASS patients meeting Connors’ criteria, 37 (45%) also met Solomon’s criteria. The mean age at time of ASS diagnosis was 60.2 ± 14.5 years and the majority of included patients (70%) were female.

Among 64 patients with ILD, 21 patients (33%) initially presented with ILD before developing other clinical manifestations associated with ASS. Among these patients, ASS clinical manifestations developed within the initial 24 months in 16 patients (76%), with the longest onset period extending to 70 months. The most common sequential ASS manifestation was mechanic’s hands (67%), followed by arthritis (43%), and Raynaud’s phenomenon (24%). Concurrent presentation of ILD and other symptoms was observed in 29 patients, whereas 14 patients maintained isolated ILD throughout the follow-up period. Notably, 6 patients presented with RP-ILD.

The diagnosis of dermatomyositis and polymyositis was established in 10 (12%) and 6 (7%) patients, respectively. A total of 24 patients (40%) had overlapping symptoms with other connective tissue diseases, including Sjogren’s syndrome in 10 (12%), SLE in 8 (10%), systemic sclerosis in 8 (10%), and rheumatoid arthritis in 7 (9%). Details of clinical manifestations and comorbidities of included patients are demonstrated in Table 1.

### 3.2. Autoantibodies

Regarding the autoantibody test, anti-PL-7 was the most frequently detected, followed by anti-Jo-1, anti-EJ, anti-PL-12, and anti-OJ, respectively (Table 1). A total of 17 patients revealed positivity for more than 1 MSAs. Of these, 2 patients demonstrated positivity for three distinct autoantibodies. Specifically, the first patient tested positive for anti-PL-7, anti-PL-12, and anti-Mi2, whereas the second patient tested positive for anti-PL-12, anti-SRP, and anti-Mi2. In total, 15 patients tested positive for two autoantibodies. Antinuclear antibodies (ANA) were tested in 70 patients, of which positive results were detected in 76% of patients. Autoantibody details are presented in Table 1 and Table 2.

Anti-Ro52 positivity was identified in 37 patients (45%). Among these, 11 patients (30%) also concomitantly exhibited positive anti-EJ, in contrast to a mere 7% among those with negative anti-Ro52 (*p* < 0.01). Furthermore, presence of anti-Ro52 demonstrated a significant associated with increased likelihood of NSIP and OP pattern, on HRCT. Specifically, NSIP was observed in 25 patients (68%) with positive anti-Ro52 compared with 19 (42%) patients without (*p* = 0.02). Similarly, OP was identified in 14 patients (38%) with positive anti-Ro52, compared with 8 (18%) in those without (*p* = 0.04).

### 3.3. Factors Associated to ILD Development

Of 82 ASS patients, 64 patients (78%) had ILD during disease course, whereas isolated ILD without other ASS clinicals was identified in 14 (17%) patients. Among 64 patients with ILD, 6 patients (9%) presented with rapid-progressive ILD (RP-ILD). The most common HRCT pattern was nonspecific interstitial pneumonia (NSIP) (69%), followed by organizing pneumonia (OP) (34%), usual interstitial pneumonia (UIP) (6%), and minimal interstitial abnormalities (5%). Regarding multivariate logistic regression, increasing age at diagnosis and the presence of anti-Ro52 were associated with increased risk of ILD, with adjusted OR of 1.10 (95% CI 1.03–1.18) and 17.92 (95% CI 2.13–138.68), respectively, whereas the presence of arthritis was associated with decreased risk of ILD (adjusted OR, 0.09; 95% CI, 0.01–0.75; *p* = 0.03), as described in Table 3.

### 3.4. Treatment

Most patients (84%) received oral prednisolone followed by hydroxychloroquine (73%), azathioprine (54%), oral cyclophosphamide (44%), mycophenolate mofetil (33%), methotrexate (20%), cyclosporine (16%), and tacrolimus (4%). Approximately 10–15% of patients received intravenous medication including cyclophosphamide (15%), methylprednisolone (12%), and rituximab (12%). When comparing ASS who had ILD to those without ILD, oral cyclophosphamide and mycophenolate mofetil were more prescribed in ASS patients with ILD, whereas hydroxychloroquine and methotrexate were more prescribed in ASS patients without ILD (Table 4).

### 3.5. Mortality

**Figure 1:**
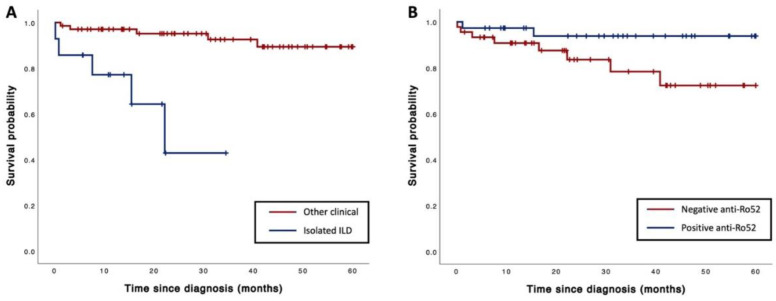
The Kaplan–Meier estimates of the survival in ASS patients categorized by (**A**) isolated ILD and (**B**) positive anti-Ro52.

With a median follow-up time of 2.5 years, mortality was observed in 10 patients (12.2%). The causes of death included fatal infection in 4 patients, advanced malignancy in 3 patients, progressive respiratory disease with respiratory failure in 2 patients, and an unknown cause in 1 patient. The factors associated with mortality are demonstrated in Table 5. Following multivariate Cox proportional hazards regression analysis, it was determined that increasing lymphocyte counts at the time of diagnosis, isolated ILD and presence of anti-Ro52 antibodies were associated with mortality, with adjusted HR levels of 0.74 (95% CI, 0.61–0.91; *p* < 0.01), 9.59 (95% CI, 1.52–60.61; *p* = 0.02) and 0.14 (95% CI, 0.02–0.93; *p* = 0.04), respectively (Table 6). The Kaplan–Meier curve illustrating the survival of ASS patients with isolated ILD and the presence of anti-Ro52 antibodies is demonstrated in Figure 1A,B.

## 4. Discussion

ASS, a subtype of IIM, is a rare autoimmune disease primarily characterized by ILD [10]. Our study demonstrated female predominance among ASS patients. ILD was the most common clinical manifestation of ASS in our study, which presented as the first manifestation in some patients. Factors associated with ILD included increasing age at diagnosis and the presence of anti-Ro52. Additionally, increasing lymphocyte counts at the time of diagnosis, isolated ILD, and the presence of anti-Ro52 antibodies were associated with mortality.

Consistent with prior studies, the most frequent clinical manifestations of ASS included ILD, arthritis, and myositis [11,12]. However, myositis was observed in only 17% of our cohort, contrasting with previous cohorts wherein myositis was detected in 60–80% of patients [11,12,13,14]. The reduced prevalence of myositis in our study correlated with the low detection of anti-Jo-1 (27%), an autoantibody associated with myositis [15].

Anti-Jo-1 antibodies were found in only 27% of our cohort, whereas other studies reported 60–80% [13,14]. However, our findings align with previous studies conducted in Asian populations, revealing that anti-Jo-1 prevalence ranging from 4 to 14% [16,17]. This might be the genetic variation in the Asian population. Furthermore, anti-Jo-1 was associated with decreased mortality [18]. Likewise, our study revealed a higher prevalence of anti-Jo-1 in survivors, although statistical significance was not attained.

Our study revealed a substantially high prevalence of ILD among ASS patients (78%) which was comparable with prior studies (61–81%) [7]. The older age at the time of diagnosis and the presence of anti-Ro52 were identified as factors associated with ILD in our study, consistent with other previous studies [19,20,21]. It was hypothesized that an underlying mechanism involving cellular aging and cellular senescence explains the correlation between older age and the development of some form of ILD [22]. However, an explicit hypothesis describing this association has not been explicitly formulated. Regarding the association between anti-Ro52 and ILD, various hypotheses have been postulated. These included the high antigenicity and widespread presence of anti-Ro52 in pulmonary tissue, as well as its presumed role in modulating host responses to viral infections, posited as a potential principal etiological factor in the onset of IIM [16].

A study conducted in China reported the prevalence of RP-ILD in 8.8% of individuals with ASS, with a higher prevalence observed in those with the anti-PL-7 subtype [23]. Similarly, our study demonstrated RP-ILD in 9.3%. However, we were unable to demonstrate specific antibodies conclusively associated with RP-ILD. The management of RP-ILD in ASS presents notable challenges. Timely recognition and intervention are essential to optimize clinical outcomes.

Regarding HRCT findings of ILD in ASS patients, the predominant radiographic pattern in our study was NSIP, apparent in 69% of all patients. OP was the second most prevalent pattern, noted in 34% of all patients, consistent with findings reported in prior studies [24]. However, no apparent association between a specific HRCT pattern and mortality was demonstrated.

The survival rate observed in our study was comparable with that previously re-ported rate [10,23]. Notably, our study revealed that patients with a positive anti-Ro52 antibody significantly improved survival outcome than those in the negative group. However, the association between the presence of anti-Ro52 and mortality remains controversial. While previous studies failed to identify an association between the presence of anti-Ro52 and mortality [21,25], a study on anti-Jo-1-positive ASS patients reported decreased survival in those with concomitant anti-Ro52 positivity [26]. Our study revealed that the presence of anti-Ro52 was associated with OP pattern and NSIP pattern in HRCT. These HRCT patterns are indicative of an inflammatory process and are known to be responsive to corticosteroid treatment. Furthermore, our study also highlighted the fact that isolated ILD was associated with an increased mortality in ASS patients. Notably, these patients tended to be male, of advanced age, and have a history of smoking.

Our study has some limitations to consider. Firstly, this study is a retrospective study, meaning that clinical ASS might not be comprehensively recorded in medical records. Secondly, we used the criteria from Connors’ for ASS diagnosis, which includes at least one clinical manifestation of ASS. Therefore, 17% of ASS patients in our study had isolated ILD without other clinical manifestations of ASS. Even though Solomon’s criteria have been proposed with the aim of more specific ASS diagnosis, some ASS patients do not have other clinical manifestations throughout the disease course [27]. Currently, the choice of criteria for ASS diagnosis remains controversial. Unfortunately, our study has a limited sample size which is unable to perform sensitivity analysis for each diagnostic criterion. Thirdly, our study was constrained by laboratory limitations, necessitating reliance on immunoblotting for MSA detection. Prior studies mentioned concerns about the comparatively higher rates of false-positive or false-negative outcomes associated with immunoblotting when compared with techniques such as immunoprecipitation or Enzyme-Linked Immunosorbent Assay (ELISA) [28].

## 5. Conclusions

ASS patients showed a favorable overall mortality rate. Variables associated with mortality risk included lymphocyte counts, the presence of anti-Ro52 antibodies, and isolated ILD. Additionally, the presence of anti-Ro52 antibodies and arthritis appeared to be associated with ILD development.

## Figures and Tables

**Table 1:** Characteristics of patients with anti-synthetase syndrome categorized by ILD.

	Total (N = 82)	ILD (N = 64)	Non-ILD (N = 18)	*p*-Value
Male, n (%)	25 (30)	23 (36)	2 (11)	0.04
Age at diagnosis, years ^§^	60.2 ± 14.5	63.3 ± 13.0	48.9 ± 13.9	<0.001
Smoking, n (%)	21 (26)	18 (28)	3 (17)	0.54
**Overlapping diseases**				
Sjogren’s syndrome, n (%)	10 (12)	7 (11)	3 (17)	0.68
SLE, n (%)	8 (10)	4 (6)	4 (22)	0.07
Systemic sclerosis, n (%)	8 (10)	8 (13)	0 (0)	0.19
Rheumatoid arthritis, n (%)	7 (9)	5 (8)	2 (11)	0.65
**Clinical features** (throughout the follow-up period)				
Arthritis, n (%)	47 (57)	32 (50)	15 (83)	0.01
Raynaud’s phenomenon, n (%)	26 (32)	19 (30)	7 (39)	0.46
Mechanic’s hands, n (%)	24 (29)	23 (36)	1 (6)	0.01
Fever, n (%)	21 (26)	17 (27)	4 (22)	1.00
Rash, n (%)	31 (38)	24 (38)	7 (39)	0.92
Oral ulcer, n (%)	4 (5)	3 (5)	1 (6)	1.00
Hair loss, n (%)	20 (24)	14 (22)	6 (33)	0.36
Sicca symptoms, n (%)	25 (31)	19 (30)	6 (33)	0.77
Weakness, n (%)	22 (27)	17 (27)	5 (28)	1.00
Myositis, n (%)	14 (17)	11 (17)	3 (17)	1.00
**Autoantibodies** (at diagnosis)				
Anti-Jo-1, n (%)	22 (27)	19 (30)	3 (17)	0.37
Anti-PL-7, n (%)	24 (29)	16 (25)	8 (44)	0.11
Anti-PL-12, n (%)	13 (16)	11 (17)	2 (11)	0.72
Anti-EJ, n (%)	14 (17)	13 (20)	1 (6)	0.29
Anti-OJ, n (%)	9 (11)	5 (8)	4 (22)	0.10
Multiple positive MSA, n (%)	17 (21)	14 (22)	3 (17)	0.75
Anti-Ro52, n (%)	37 (45)	34 (53)	3 (17)	0.01
Antinuclear antibodies, n (%) ^†^	53 (76)	42 (76)	11 (73)	1.00
-Speckle pattern, n (%)	30 (57)	23 (55)	7 (64)	0.74
-Homogeneous pattern, n (%)	16 (30)	12 (29)	4 (36)	0.72
-Nucleolar pattern, n (%)	13 (25)	8 (19)	5 (46)	0.11
-Multiple nuclear dot pattern, n (%)	7 (23)	7 (30)	0 (0)	0.15
-Centromere pattern, n (%)	2 (4)	2 (5)	0 (0)	1.00
-Cytoplasmic staining pattern, n (%)	17 (24)	14 (25)	3 (20)	1.00
**Laboratory data** (at diagnosis)				
ESR, mm/hour ^¶^	35 (20–62)	35.5 (20–58)	33 (18–68)	0.87
Creatine kinase, IU/L ^¶^	90 (60–153)	91 (61–201)	87 (50–103)	0.21
C-reactive protein, mg/dL ^¶^	5.9 (1.1–18.9)	6.2 (1.2–9.1)	3.4 (0.6–16.1)	0.40
Hemoglobin levels, g/dL ^§^	12.1 ± 1.8	12.3 ± 1.7	11.6 ± 2.0	0.16
White blood cell count, 10^9^/L ^¶^	7.9 (5.7–10.6)	8.5 (6.3–11.5)	6.3 (5.5–7.9)	0.01
Platelet count, 10^9^/L ^¶^	288 (216–358)	290 (218–360)	282 (212–354)	0.50
**Underlying diseases**				
Hypertension, n (%)	35 (43)	32 (50)	3 (17)	0.01
Diabetes mellitus, n (%)	20 (24)	14 (22)	6 (33)	0.32
COPD, n (%)	5 (6)	5 (8)	0 (0)	0.22
Cerebrovascular disease, n (%)	3 (4)	3 (5)	0 (0)	0.35
Coronary artery disease, n (%)	9 (11)	7 (11)	2 (11)	0.98
Cirrhosis, n (%)	3 (4)	3 (5)	0 (0)	0.35
Malignancy, n (%)	10 (12)	8 (13)	2 (11)	0.87

^§^ Data report as mean ± SD. ^†^ Total 70 patients tested ANA. ^¶^ Data reported as median (interquartile range). COPD, chronic obstructive pulmonary disease; ESR, erythrocyte sedimentation rate; ILD, interstitial lung disease; SLE, systemic lupus erythematosus.

**Table 2:** Details of MSAs among 15 ASS patients who tested positive for 2 MSAs.

	Anti-Jo-1	Anti-PL-7	Anti-PL-12	Anti-EJ	Anti-OJ
**Anti-Jo-1**					
**Anti-PL-7**	0				
**Anti-PL-12**	0	0			
**Anti-EJ**	0	1	0		
**Anti-OJ**	0	0	0	1	
**Anti-SRP**	1	0	0	0	0
**Anti-Mi2**	2	3	3	0	1
**Anti-MDA5**	0	0	0	1	0
**Anti-TIF1γ**	0	1	1	0	0

**Table 3:** Factors associated with ILD by multivariate logistic regression analysis.

	Adjusted Odds Ratio (95% CI)	*p*-Value
Age at diagnosis	1.10 (1.03–1.18)	0.01
Arthritis	0.09 (0.01–0.75)	0.03
Positive anti-Ro52	17.92 (2.13–138.68)	0.01
History of hypertension	7.27 (2.13–138.68)	0.06
Mechanic’s hands	13.05 (0.72–235.38)	0.08

Multivariate logistic regression model using backward method was adjusted by age, sex, history of SLE, arthritis, mechanic’s hands, anti-OJ antibody, anti-Ro-52, white blood cell count, and history of hypertension. Variables, adjusted OR, and *p*-values shown in the table were the remaining variables in the last step of the multivariate logistic regression model.

**Table 4:** Treatment of ASS categorized by the presence of ILD.

	Total (N = 82)	ILD (N = 64)	Non-ILD (N = 18)	*p*-Value
Oral prednisolone	69 (84)	54 (84)	15 (83)	1.00
Hydroxychloroquine	60 (73)	42 (67)	18 (100)	<0.01
Azathioprine	44 (54)	33 (52)	11 (61)	0.60
Oral cyclophosphamide	36 (44)	33 (52)	3 (17)	<0.01
Mycophenolate mofetil	27 (33)	25 (39)	2 (11)	0.03
Methotrexate	16 (20)	9 (14)	7 (39)	0.04
Cyclosporine	13 (16)	11 (17)	2 (11)	0.72
Intravenous cyclophosphamide	12 (15)	10 (16)	2 (11)	1.00
Intravenous methylprednisolone	10 (12)	8 (13)	2 (11)	1.00
Rituximab	10 (12)	10 (16)	0 (0)	0.11
Tacrolimus	3 (4)	3 (5)	0 (0)	1.00

**Table 5:** Association of clinical characteristics with all-cause mortality.

	Death (N = 10)	Survival (N = 72)	HR (95% CI)	*p*-Value
Male, n (%)	4 (40)	21 (29)	1.6 (0.5–5.8)	0.45
Age at diagnosis, years ^§^	66.9 ± 10.5	59.2 ± 14.7	1.05 (0.90–1.10)	0.06
Smoking, n (%)	6 (60)	15 (21)	4.4 (1.3–15.8)	0.02
**Overlapping diseases**				
Rheumatoid arthritis, n (%)	0 (0)	7 (10)	0.04 (0–707)	0.33
Sjogren’s syndrome, n (%)	1 (10)	9 (13)	0.6 (0.1–4.8)	0.64
SLE, n (%)	1 (10)	7 (10)	0.9 (0.1–7.5)	0.95
Systemic sclerosis, n (%)	1 (10)	7 (10)	1.2 (0.2–9.8)	0.84
**Clinical features**				
ILD, n (%)	7 (70)	57 (79)	0.7 (0.2–2.8)	0.66
Rapid progressive, n (%)	2 (20)	4 (6)	3.5 (0.7–16.4)	0.11
Isolated ILD, n (%)	5 (50)	9 (13)	10.9 (2.8–43.2)	<0.01
Arthritis, n (%)	4 (40)	43 (60)	0.4 (0.1–1.3)	0.13
Raynaud’s phenomenon, n (%)	2 (20)	24 (33)	0.5 (0.1–2.3)	0.36
Mechanic’s hands, n (%)	0 (0)	24 (33)	0.03 (0–6.3)	0.19
Fever, n (%)	2 (20)	19 (26)	0.5 (0.1–2.6)	0.45
Rash, n (%)	3 (30)	28 (39)	0.5 (0.1–2.1)	0.36
Oral ulcer, n (%)	0 (0)	4 (6)	0.1 (0–19,116)	0.64
Hair loss, n (%)	1 (10)	19 (26)	0.3 (0.1–2.3)	0.25
Sicca symptoms, n (%)	2 (20)	23 (32)	0.8 (0.2–3.7)	0.76
Weakness, n (%)	3 (30)	19 (26)	1.1 (0.3–4.1)	0.92
**Autoantibodies**				
Anti-Jo-1, n (%)	0 (0)	22 (31)	0.03 (0–14.7)	0.27
Anti-PL-7, n (%)	5 (50)	19 (26)	2.4 (0.7–8.4)	0.16
Anti-PL-12, n (%)	3 (30)	10 (14)	2.4 (0.7–9.3)	0.21
Anti-EJ, n (%)	1 (10)	13 (18)	0.4 (0.1–3.3)	0.40
Anti-OJ, n (%)	1 (10)	8 (11)	0.8 (0.1–6.5)	0.85
Multiple positive MSA, n (%)	4 (40)	13 (18)	2.8 (0.8–9.9)	0.11
Anti-Ro52, n (%)	2 (20)	35 (49)	0.3 (0.1–1.2)	0.08
Antinuclear antibodies, n (%) ^†^	7 (78)	46 (75)	1.3 (0.3–6.2)	0.76
-Speckle pattern, n (%)	3 (43)	27 (59)	0.5 (0.1–2.4)	0.42
-Homogeneous pattern, n (%)	3 (43)	13 (28)	1.6 (0.4–7.4)	0.53
-Nucleolar pattern, n (%)	3 (43)	10 (22)	2.2 (0.5–10.2)	0.31
-Multiple nuclear dot pattern, n (%)	0 (0)	7 (27)	0.03 (0–490)	0.48
-Centromere pattern, n (%)	1 (14)	1 (2)	12.0 (1.1–132)	0.04
-Cytoplasmic staining pattern, n (%)	2 (22)	15 (25)	0.6 (0.1–2.9)	0.49
**Laboratory data (at diagnosis)**				
ESR, mm/hour ^¶^	53 (27–67)	34 (20–62)	1.01 (0.99–1.03)	0.41
C-reactive protein, mg/dL ^¶^	17.5 (9.9–68.2)	4.3 (1.0–17.6)	1.01 (0.99–1.03)	0.11
Creatine kinase, IU/L ^¶^	50 (20–69)	93 (68–164)	0.99 (0.98–1.01)	0.26
Hemoglobin levels, g/dL ^§^	10.5 ± 1.5	12.3 ± 1.8	0.6 (0.4–0.9)	< 0.01
White blood cell count, 10^9^/L ^¶^	7.5 (5.8–11.0)	8.0 (5.8–11.2)	0.9 (0.8–1.1)	0.37
Lymphocyte count, 10^9^/L	0.9 (0.4–1.3)	1.8 (1.3–2.5)	0.2 (0.1–0.6)	<0.01
Platelet count, 10^9^/L ^¶^	234 (163–384)	290 (217–357)	0.99 (0.98–1.00)	0.21
**HRCT pattern**				
Emphysema, n (%)	3 (30)	3 (4)	7.4 (1.9–29.1)	<0.01
Evidence of air trapping, n (%)	2 (20)	22 (31)	0.8 (0.2–4.1)	0.82
NSIP, n (%)	6 (60)	38 (53)	1.3 (0.4–4.5)	0.72
Honeycombing, n (%)	1 (10)	6 (8)	1.3 (0.2–10.3)	0.80
UIP or probable UIP, n (%)	0 (0)	6 (8)	0.04 (0–767)	0.53
OP, n (%)	3 (30)	19 (26)	1.1 (0.3–4.4)	0.84
**Underlying disease**				
Hypertension, n (%)	8 (80)	27 (38)	6.8 (1.4–32.2)	0.02
Diabetes mellitus, n (%)	6 (60)	14 (19)	6.0 (1.7–21.5)	<0.01
COPD, n (%)	2 (20)	3 (4)	4.1 (0.9–19.4)	0.07
Cerebrovascular disease, n (%)	0 (0)	3 (4)	0.05 (0–>10,000)	0.77
Coronary artery disease, n (%)	1 (10)	8 (11)	0.99 (0.13–7.87)	1.00
Cirrhosis, n (%)	2 (20)	1 (1)	9.6 (2.0–45.4)	<0.01
Malignancy, n (%)	6 (60)	4 (6)	21.5 (5.1–89.7)	<0.01

^§^ Data report as mean ± SD. ^†^ Total 70 patients tested ANA. ^¶^ Data report as median (interquartile range). COPD, chronic obstructive pulmonary disease; ESR, erythrocyte sedimentation rate; ILD, interstitial lung disease; MSA, Myositis-specific antibodies; NSIP, non-specific interstitial pneumonia; OP, organizing pneumonia; SLE, systemic lupus erythematosus; UIP, usual interstitial pneumonia.

**Table 6:** Factors associated with all-cause mortality via Multivariate Cox-proportional-hazard regression.

	Adjusted Hazard Ratio (95% CI)	*p*-Value
Increasing lymphocyte counts per 100 × 10^9^/L	0.74 (0.61–0.91)	<0.01
Isolated ILD	9.59 (1.52–60.61)	0.02
Presence of anti-Ro52	0.14 (0.02–0.93)	0.04
History of cirrhosis	8.55 (0.95–77.08)	0.06

Multivariable Cox proportional-hazards regression using backward method was adjusted by age, smoking history, isolated ILD, anti-Ro52, presence of emphysema in HRCT, hemoglobin levels, lymphocyte count, history of DM, history of cirrhosis, and history of solid malignancy. Variables, adjusted HR, and *p*-values which demonstrated in the table were remaining variables in the last step of the model.

## Data Availability

Data is unavailable due to privacy and ethical restrictions.
